# Perceptions of Visualizing Physical Activity as a 3D-Printed Object: Formative Study

**DOI:** 10.2196/12064

**Published:** 2019-01-30

**Authors:** Sam Graeme Morgan Crossley, Melitta Anne McNarry, Joanne Hudson, Parisa Eslambolchilar, Zoe Knowles, Kelly Alexandra Mackintosh

**Affiliations:** 1 Applied Sport Technology Exercise and Medicine Research Centre School of Sport and Exercise Sciences Swansea University Swansea United Kingdom; 2 Human Factors Technology Research Priority Area School of Computer Science and Informatics Cardiff University Cardiff United Kingdom; 3 Physical Activity Exchange School of Sport and Exercise Sciences Liverpool John Moores University Liverpool United Kingdom

**Keywords:** 3D printing, feedback, youth, education, school

## Abstract

**Background:**

The UK government recommends that children engage in moderate-to-vigorous physical activity for at least 60 min every day. Despite associated physiological and psychosocial benefits of physical activity, many youth fail to meet these guidelines partly due to sedentary screen-based pursuits displacing active behaviors. However, technological advances such as 3D printing have enabled innovative methods of visualizing and conceptualizing physical activity as a tangible output.

**Objective:**

The aim of this study was to elicit children’s, adolescents’, parents’, and teachers’ perceptions and understanding of 3D physical activity objects to inform the design of future 3D models of physical activity.

**Methods:**

A total of 28 primary school children (aged 8.4 [SD 0.3] years; 15 boys) and 42 secondary school adolescents (aged 14.4 [SD 0.3] years; 22 boys) participated in semistructured focus groups, with individual interviews conducted with 8 teachers (2 male) and 7 parents (2 male). Questions addressed understanding of the physical activity guidelines, 3D model design, and both motivation for and potential engagement with a 3D physical activity model intervention. Pupils were asked to use Play-Doh to create and describe a model that could represent their physical activity levels (PAL). Data were transcribed verbatim and thematically analyzed, and key emergent themes were represented using pen profiles.

**Results:**

Pupils understood the concept of visualizing physical activity as a 3D object, although adolescents were able to better analyze and critique differences between low and high PAL. Both youths and adults preferred a 3D model representing a week of physical activity data when compared with other temporal representations. Furthermore, all participants highlighted that 3D models could act as a motivational tool to enhance youths’ physical activity. From the Play-Doh designs, 2 key themes were identified by pupils, with preferences indicated for models of abstract representations of physical activity or bar charts depicting physical activity, respectively.

**Conclusions:**

These novel findings highlight the potential utility of 3D objects of physical activity as a mechanism to enhance children’s and adolescents’ understanding of, and motivation to increase, their PAL. This study suggests that 3D printing may offer a unique strategy for promoting physical activity in these groups.

## Introduction

### Background

The UK government recommends that children aged 5 to 18 years engage in at least 60 min of moderate-to-vigorous physical activity (MVPA) every day [1]. Despite the well-established physiological and psychosocial health benefits of regular physical activity for youths [2-5], many fail to meet these recommended guidelines [6]. More specifically, for these populations, sedentary screen-based pursuits are thought to have displaced active behaviors and have been independently associated with adverse health outcomes such as obesity [7] and hypertension [8]. According to Noonan et al [9], there is a lack of understanding within youths on the various forms of physical activity, including those of active travel and unstructured play, with a need to educate how these types of activities contribute to achieving the physical activity recommendations. Conversely, Kremers et al [10] argue that a lack of awareness of physical activity among youths is likely to make them less susceptible to educational programs that are aimed to influence attitudes, norms, self-efficacy, or other cognitive means, as they will not perceive the need to change. Indeed, research supports this notion, demonstrating that youths who are aware of their physical activity levels (PAL) and the recommended guideline are, on average, 20 min more active than their unaware counterparts [10], and as a result, they are more likely to achieve the daily 60 min of MVPA [11,12]. Therefore, developing youths’ understanding and awareness of their physical activity behaviors is crucial for implementing a successful health program designed to increase PAL [10].

Schools have been identified as ideal settings to integrate health-promoting interventions because of their established infrastructure and role in health education [13]. Subsequently, researchers have developed numerous school-based interventions that seek to utilize technology as part of the solution, rather than part of the problem [14-18]. Although technology-based interventions have shown promise in improving psychosocial outcomes, efforts to elicit sustainable behavior change have been less consistent [19]. This may, at least in part, be a result of the traditional power structure of the *all-knowing* adult and the *all-learning* child [20], where adults’ development of new technology limits the personal opinions of youths when it comes to deciding what technology should be used within a school-based environment [20].

To develop a successful physical activity intervention, an appropriate conceptual health promotion model should be utilized to focus on the most salient characteristics of the target group [21]. One such model, which is specifically relevant to children’s physical activity, is the Youth Physical Activity Promotion Model (YPAPM) [22]. This model provides a comprehensive and structured assessment of the target population’s own needs and barriers to participation in physical activity, acknowledging children as the experts [23], and allowing intervention design through the eyes of the child rather than the researcher, teacher, or parent [9]. As argued by Druin [20], children as design partners can play an impactful role in the creation of new technologies that are not only going to be effective and meaningful but also will excite children and aid learning.

Research shows that 80% of youths are visual and tactile learners [24]; therefore, relying simply on numbers and figures as a source of knowledge is limited [25], and richer ways of data representation are required [26]. Indeed, visualizations can play a key role in motivating individuals to enhance their PAL, enabling reflection on personal performance and current level of physical activity [27]. A recent school-based intervention using glanceable light-emitting diode (LED) technology to display groups’ PAL reported that children wanted more personalized forms of visual feedback [18], with others suggesting that material rewards are cherished more than virtual rewards [28] because of their higher visibility and uniqueness [29,30]. Indeed, previous research utilizing paper and LED lights to create PA awareness promoting artifacts found that youth took incremental steps toward self-regulation through goal setting and reflection [31]. It was concluded that although the artifacts did not elicit improved physical activity in youth, using tangible artifacts in conjunction with wearables could benefit youths’ health [31]. However, it could be argued that paper artifacts do not provide youth with an adequate haptic and proprioceptive experience of personalized feedback to reap health benefits [32]. With the recent rise of the *maker movement* and cost-effective 3D printers [33], numerous opportunities in health-related research have emerged, utilizing 3D printers to create tangible visualizations of physical activity [34-36]. As Jansen et al [37] advocate, there are many benefits of tangible visualizations over on-screen visualizations of data, which include (1) allowing for a more active perception, (2) leveraging nonvisual senses such as touch, (3) integration with the physical world, and (4) harnessing the interplay between vision and touch to facilitate cognition. For example, Khot et al [38] transformed adults’ heart rate data into 3D-printed artifacts, with participants reporting that the artifacts acted as a reward and allowed reflection and reminiscence on past physical activities [38]. Indeed, tangible interfaces have been reported to involve children in playful learning [39], engagement, and reflection [40]. Consistent with goal-setting theory [41], incentives are important in maintaining interest in an activity, with incentive-based interventions to *nudge* healthy behavior change in youths demonstrating potential [42,43]. However, whether personalized 3D-printed objects can be used to enhance youths’ understanding, awareness, and motivation relating to engagement in physical activity remains to be elucidated.

### Aims

Therefore, the aims of the present study were to (1) formatively elicit children’s, adolescents’, teachers’, and parents’ perceptions of physical activity data when represented as 3D-printed objects; (2) ascertain how youths visualize their personal 3D objects of physical activity using Play-Doh; (3) obtain parents’ and teachers’ views on the perceived benefits and barriers of 3D-printed objects of physical activity for youths; and (4) use these data to subsequently inform the design of 3D models and a school-based physical activity intervention.

## Methods

### Recruitment

In total, 20 primary and secondary schools from the Swansea region of South Wales were contacted and invited to take part. The schools were stratified into high and low socioeconomic status (SES) according to the percentage of students per school eligible to receive free school meals [44]. From those schools that expressed an interest (35%, 7/20) response rate), 4 schools, 1 high- and 1 low-SES primary and secondary schools, were selected based on order of availability to take part in the study. Overall, 27 primary school children (aged 8.4 [SD 0.3] years; 15 boys) and 42 secondary school adolescents (aged 14.4 [SD 0.3]; 22 boys), 8 teachers (2 male), and 7 parents (2 male) provided written informed parental or carer consent and child assent, as appropriate, to participate in the study. All procedures were approved by the Swansea University A-STEM Ethics Committee and were conducted in accordance with the Declaration of Helsinki (reference number: PG/2014/40).

### Procedures

All semistructured focus group discussions and interviews were conducted by the first author (SGMC) in a nondirective and unbiased way [45], with 6 groups of children, 8 groups of adolescents, and a total of 13 individual interviews with teachers and parents. Sample questions for the focus groups and one-to-one interviews are presented in Table 1. On 2 separate occasions, 2 parents and 2 teachers were interviewed together because of restricted availability [46]. Focus group discussions with youths involved 4 to 6 participants to allow for lively, yet manageable, interactions [45,47,48], with the exception of 1 primary school focus group where a child with special educational needs required a smaller group of 3 children with 1 support teacher. Both single- and mixed-sex focus groups were conducted [49]. All focus group sessions were completed within the school environment, either within a familiar classroom or in the school library, to provide comfort and reduce anxiety [50]. Participants were seated in a circular arrangement around a table to create a relaxed and informal atmosphere [45], maximizing social interaction and observer involvement [51]. Moreover, this seating arrangement allows the facilitator to sit among the participants to establish a nonauthoritarian approach to questioning. To ensure each of the group members was comfortable with talking aloud and to create an environment in which sharing and listening were valued, an icebreaker question was used [52]. The semistructured focus group questions were informed by enabling, reinforcing, and predisposing factors from the YPAPM [22] to explore physical activity engagement and identify any barriers toward 3D-printed objects in an age-appropriate manner. All predetermined questions were reviewed and discussed by SGMC, MAM, PE, and KAM, and additional feedback was provided independently by 2 Health and Care Professions Council–registered practitioner psychologists (JH and ZK).

Alongside focus group discussions and one-to-one interviews, children, adolescents, and adults were all shown a custom-made video on the concept of 3D printing physical activity. Following this, participants were shown 3 different prototype 3D-printed models displaying example accelerometry-derived physical activity data, and discussions focused on how participants thought the physical activity data were represented by these models. Finally, children and adolescents were asked to independently design their own personalized model of physical activity using Play-Doh. The Play-Doh modeling process builds on the principles of the write, draw, show and tell method [9] by replacing the write and draw components of the framework with the modeling of Play-Doh. Following the Play-Doh modeling task, the facilitator asked each child to articulate and explain the characteristics of their design in a verbal statement at their own pace. All Play-Doh models were photographed for further analyses.

Focus group discussions lasted between 60 and 90 min and 50 and 60 min for primary and secondary school groups, respectively, and adult interviews lasted approximately 25 to 45 min. All the focus groups and one-to-one interviews were digitally voice-recorded (Olympus DM-520 Digital Voice Recorder; Shinjuku, Japan) and video-recorded (Sony Handycam HDR-PJ540, Minato, Japan).

### Data Analysis

All focus group discussions and one-to-one interviews were transcribed verbatim, resulting in 774 pages (327, 297, and 150 pages for children, adolescents, and adults, respectively) of raw data. Researchers SGMC, MAM, and KAM read each transcript to familiarize themselves with the data. Transcripts were thematically analyzed by SGMC using data coding and identification of themes [53]. Transcripts were first deductively analyzed using aspects of the YPAPM as a thematic framework [22].

**Table 1:** Example focus group and interview questions.

Interview	Topic	Example
Children and adolescents	Motivation	What would you think if I said we could 3D print your own personal model, which shows how physically active you are?
Children and adolescents	Model design	What sort of model would you like to develop or represent your own physical activity as in the video, how would it look?
Adults	Motivation	How do you think the 3D models of physical activity could motivate children to be more physically active?
Adults	Model design	Are there any models that you think would be good to help children to visualize physical activity?

Additional emergent themes were then further explored using an inductive process. Both deductive and inductive processes used a manual cut-and-paste technique to identify key themes. Participants’ verbatim quotations were chosen by SGMC and discussed in collaboration with MAM and KAM. A frequency count for the meaningful quotes was conducted to record how many participants responded within emergent themes. The themes, meaningful quotations, and frequency counts were then displayed diagrammatically using a pen profile approach. Pen profiling has been used within studies exploring perceptions and experiences of physical activity in youths [47,54] and is considered to be an accessible technique for researchers who have both quantitative and qualitative backgrounds [55]. Through the process of reverse triangulation, authors critically questioned and cross-examined the data in reverse from the pen profiles to the transcripts. This process was repeated, allowing authors to offer alternative interpretations of the data, until a consensus was reached to finalize the pen profile designs. In some cases, visual illustrations were presented to add more context to the data collected. Triangulation of the data tests the robustness of the findings and ensures methodological rigor using a *trustworthiness criterion* [56]. The criterion places trust in the researcher responsible for data collection to determine key findings that are worthy of attention. These were then assessed by PE, JH, and ZK who were not as directly involved in the analysis process [57]. In addition, the primary and secondary school participants’ Play-Doh model photos aligned with the relevant verbal statements were analyzed by SGMC, MM, JH, PE, and KAM as a group to identify common trends and designs. Specifically, all Play-Doh model photos, with their respective verbal statements, were displayed on a large white board and appraised by the research team. Throughout this process, the Play-Doh models were grouped based on similar structural (eg, sun or bar chart design) and verbal (eg, the more physical activity you do, the larger the model) characteristics. The most common Play-Doh model designs created by children (abstract, 12/28; graphical, 15/28) and adolescents (graphical, 28/42) were subsequently considered for further interpretation and 3D model design.

## Results

### Perceptions and Designs of 3D Physical Activity Models

**Figure 1:**
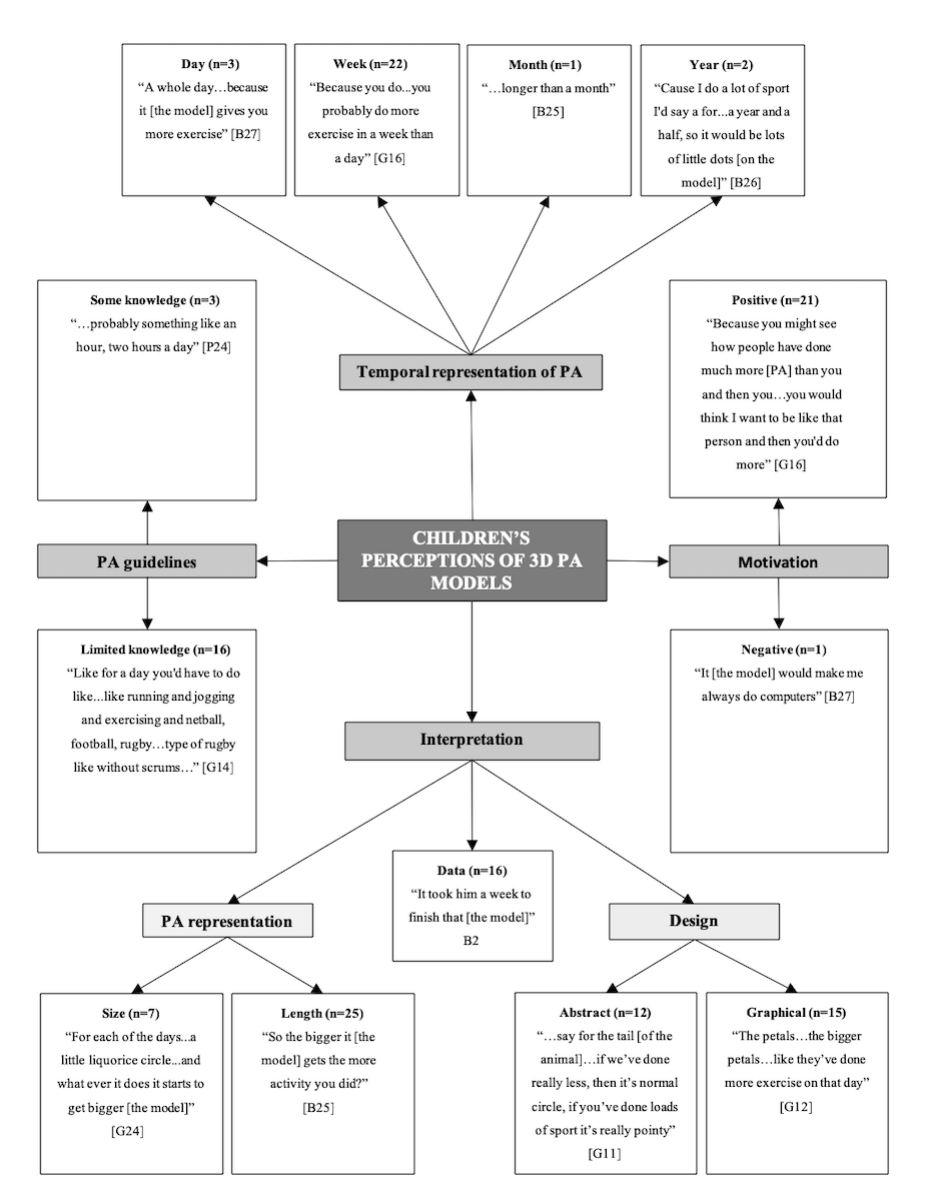
Children’s pen profile. B: boy; G: girl; PA: physical activity; n: frequency counts.

**Figure 2:**
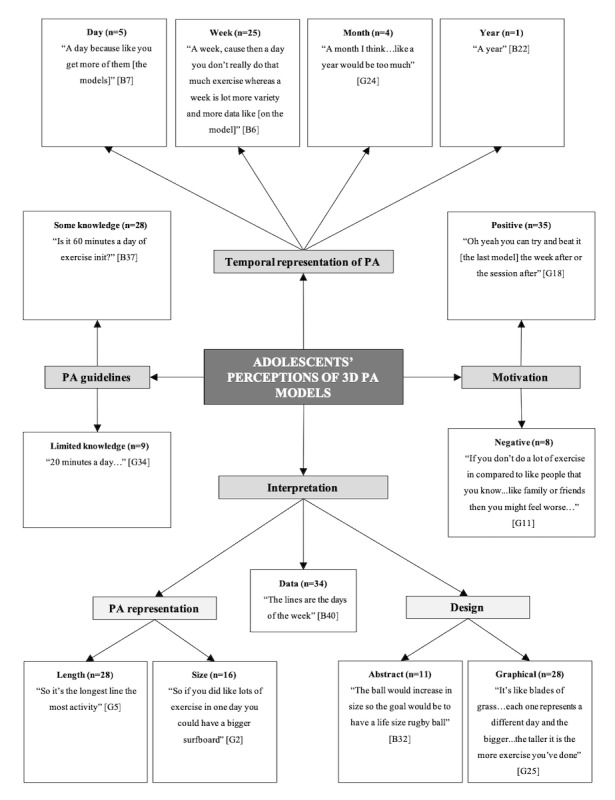
Adolescents’ pen profile. B: boy; G: girl; PA: physical activity; n: frequency counts.

**Figure 3:**
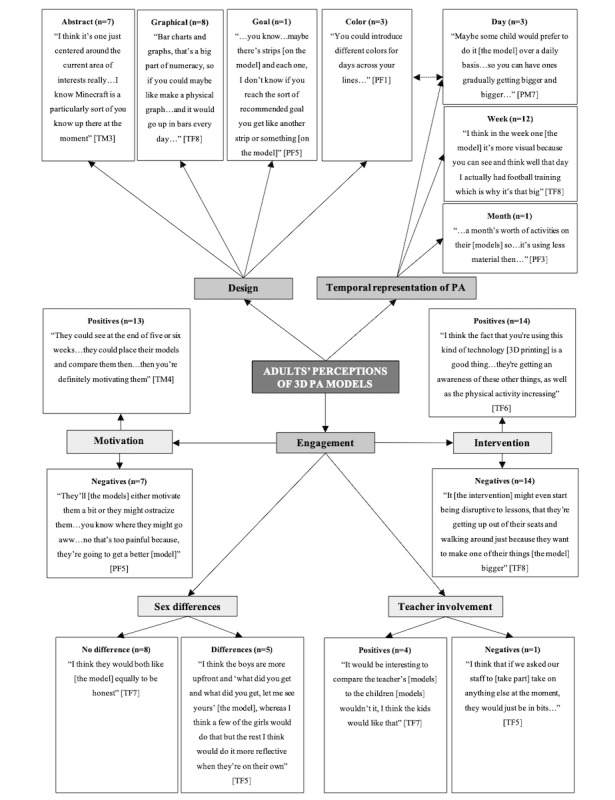
Adults’ pen profile. T: teacher; P: parent; M: male; F: female; PA: physical activity; n: frequency counts.

In total, 3 separate pen profiles were constructed to represent children’s (Figure 1), adolescents’ (Figure 2), and combined parents’ and teachers’ (Figure 3) perceptions of 3D models. There were consistent themes identified between parents and teachers, and therefore, their data were combined for final analysis.

### Children’s Perceptions and Designs of 3D Physical Activity Models

As shown in Figure 1, key emergent themes were structured around “Temporal Representation of Physical Activity,” “Motivation,” “Interpretation,” and “Physical Activity Guidelines.” The higher order theme “Interpretation” was linked to further subthemes “Physical Activity Representation” and “Design.” Primary school children demonstrated the ability to interpret and apply the different component lengths and sizes of the prototype 3D models in relation to physical activity parameters. Specifically, 92% (25/28) of children were able to accurately understand how the changing length of the model represented increasing levels of physical activity. However, only 26% (7/28) of children were able to understand the alternative method of increasing the size of the model to represent greater levels of physical activity. The physical activity data displayed on the models were correctly identified by 59% (16/28) of the children as representing either hours or days of physical activity. The majority of children (81%, 22/28) preferred the 3D models to represent a week of their physical activity data, compared with a day (3/28, 11%), year (2/28, 7%), or month (1/28, 4%):

Because you do...you probably do more exercise in a week than a day.

G16

**Figure 4:**
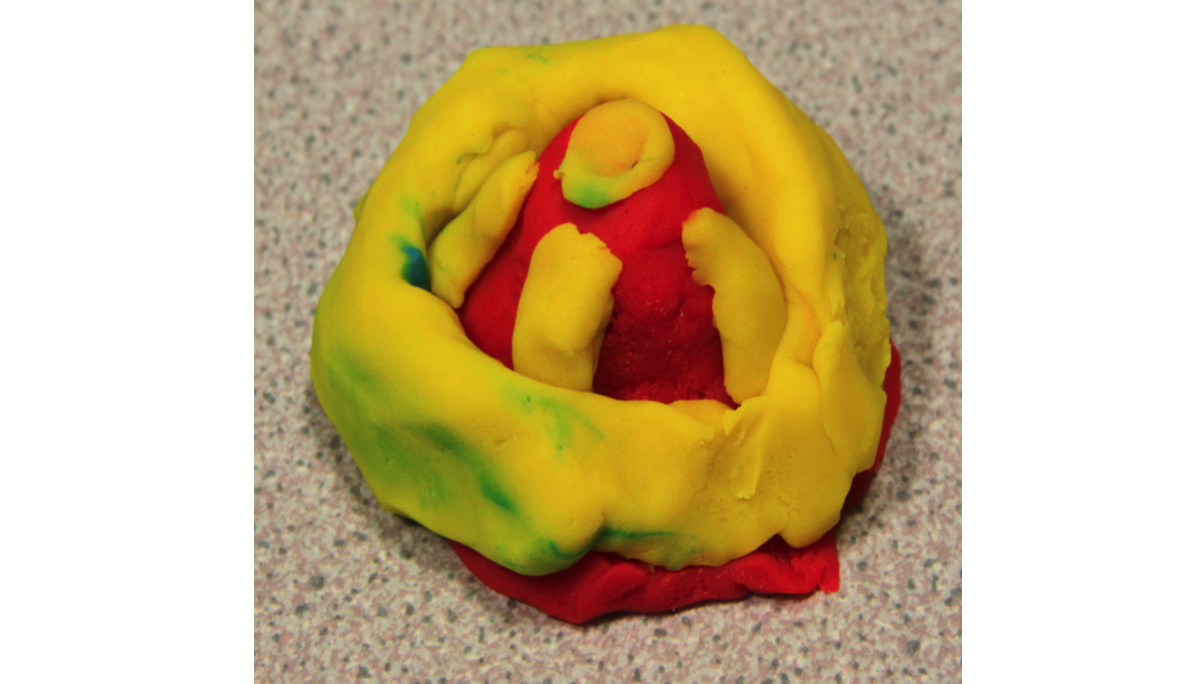
Child's abstract volcano model design.

**Figure 5:**
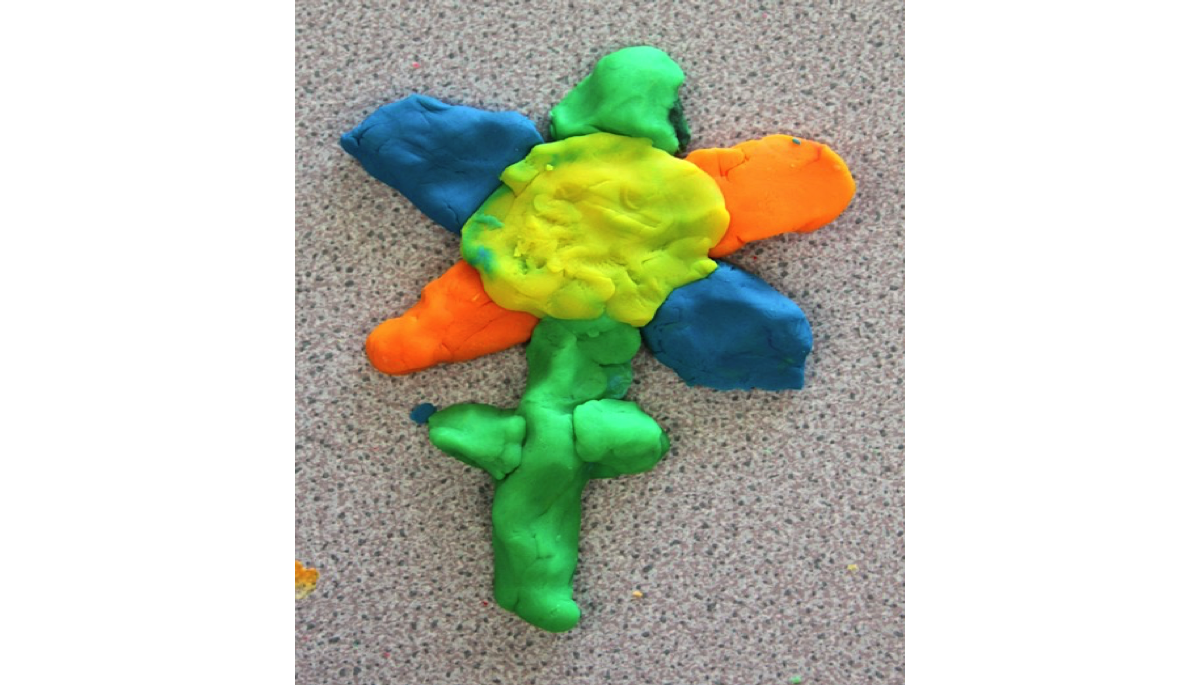
Child's graphical flower model design.

From the Play-Doh modeling task, 2 subthemes emerged, one being *abstract* and the other *graphical*. Children revealed no preference for abstract (12/28, 44%) or graphical (15/28, 56%) model representations of physical activity. Children’s abstract models were characterized by the model changing shape or size, such as a volcano with more lava erupting for higher levels of physical activity (Figure 4). Graphical representations, such as the flower (Figure 5), distinguished between different hours, days, or weeks of physical activity completed (ie, the flower’s petals resembling the different days of physical activity).

A total of 21 children (78%, 21/28) commented that the 3D models had potential to motivate themselves to engage in more physical activity, substantially outweighing the negatives expressed by 1 child. Specifically, children revealed that the 3D models would add competition between classmates and motivate them to do more. For example:

Because you might see how people have done much more [physical activity] than you and then you...you would think I want to be like that person and then you’d do more.

G16

Overall, 16 children (59%) displayed limited knowledge of the current UK government physical activity guidelines or how to achieve them, with only 3 children able to express the amount through the context of time spent being physically active, with no reference to intensity level. For example:

...probably something like an hour, two hours a day...

PL9

### Adolescents’ Perceptions and Designs of 3D Physical Activity Models

Overall, 4 higher order themes were identified structured around “Temporal representation,” “Motivation,” “Interpretation,” and “Physical activity guidelines” (Figure 2). The higher order theme *Interpretation* was further linked to subthemes *Physical activity representation* and *design*. Adolescents demonstrated the ability to identify and compare the different components of the prototype 3D models and their changing length and size in relation to physical activity. Specifically, the increasing size (16/42, 38%) and length (28/42, 67%) of the models were correctly interpreted as representing higher PAL. The majority (34/42, 81%) of adolescents showed a clear understanding of the represented data on the models. For example:

The lines [on the models] are the days of the week.

B30

...so does that mean he’s most active Tuesday, Wednesday, Thursday sort of thing.

G3

**Figure 6:**
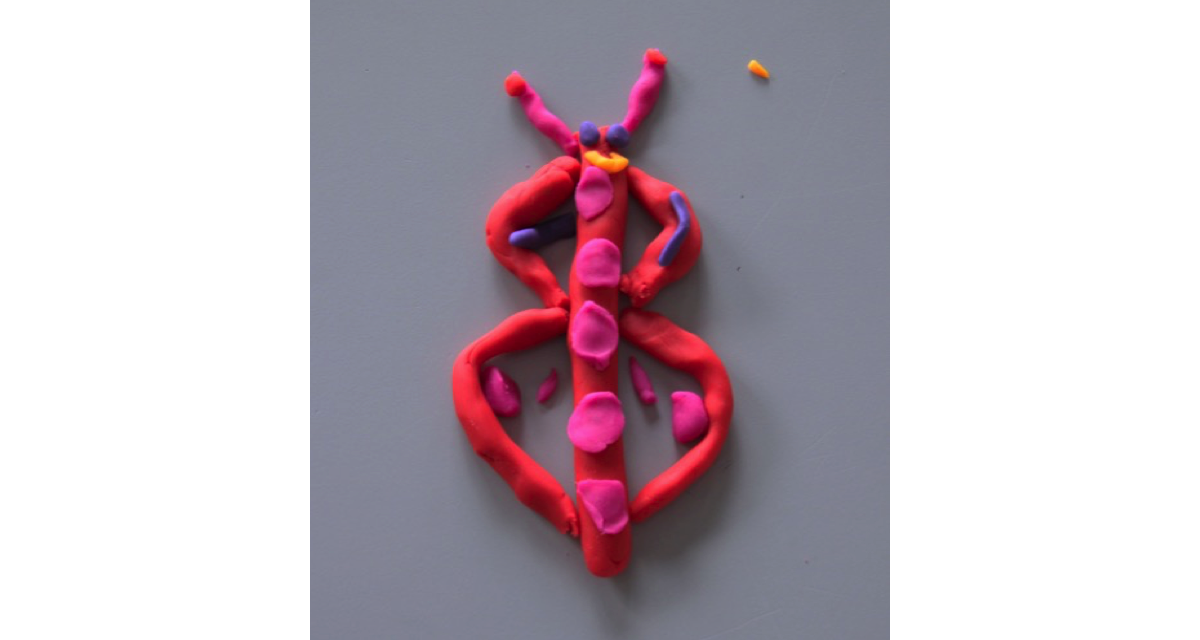
Adolescent's abstract butterfly model design.

**Figure 7:**
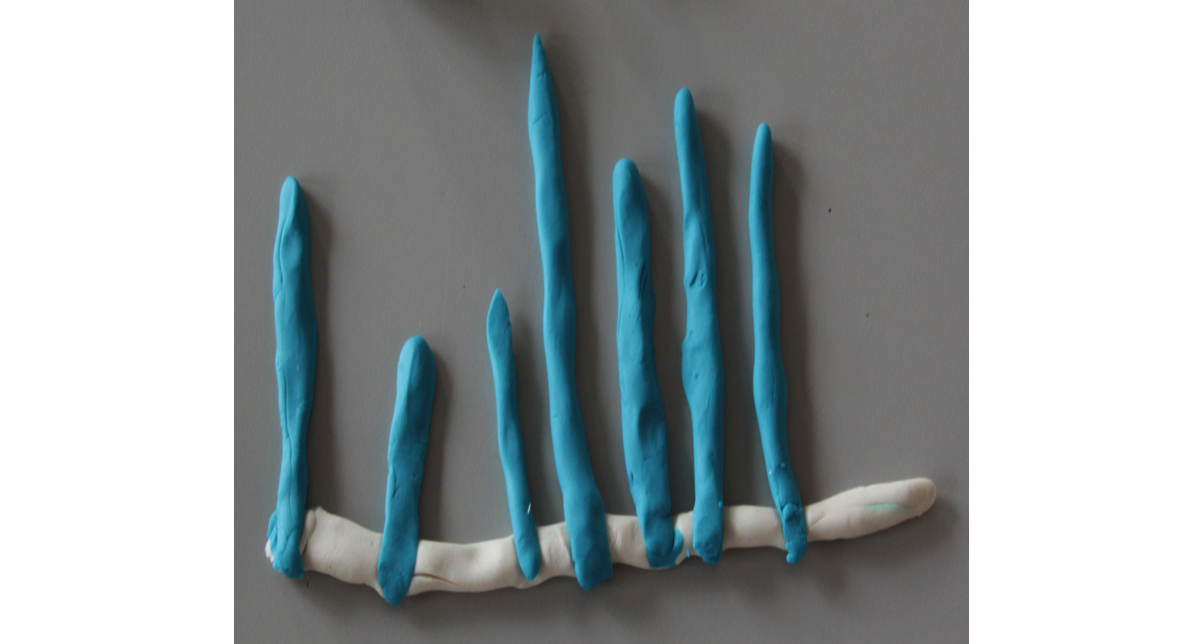
Adolescent's graphical bar chart model design.

Adolescents highlighted a preference for a week (25/42, 60%) of physical activity data to be displayed on the 3D models because of the greater variety and reflection of their PAL in a week when compared with a model based on a day (5/42, 12%), month (4/42, 10%), or year (1/42, 2%). The Play-Doh modeling task displayed similar subthemes to those found in children, with a larger proportion of designs displaying graphical (28/42, 67%) compared with abstract (11/42, 26%) designs. Abstract models, such as the butterfly (Figure 6), were characterized by the changing size or detail of the models. Graphical representations resembled typical bar charts or line graphs (Figure 7) to display different days, weeks, or months of physical activity.

A total of 35 adolescents (83%, 35/42) expressed that the 3D models would motivate them to engage in more physical activity by beating previous models. For example:

Oh yeah you can try and beat it [the model] the week after or the session after.

G18

Overall, 8 adolescents (19%, 8/42) thought that the 3D models may discourage engagement in physical activity because of feelings of doing worse than others and embarrassment if the model showed low PAL:

If you don’t do like a lot of exercise in compared to like people that you know...like family or friends then you might feel worse...

G11

...if other people like saw the object or something it might be a bit embarrassed if you haven’t done enough exercise.

G21

In total, 28 adolescents (67%, 28/42) showed some knowledge of the government guidelines for physical activity. A specific Sport Wales initiative called *5x60* [58] may have influenced these findings:

...they [Sport Wales officers] try and get everyone to do five sessions of sixty minutes a week of exercise.

G3

...yeah what’s it called...five sixty...five hours of sixty...no five lots of sixty minutes per week...

G18

### Adults’ Perceptions and Designs of 3D Physical Activity Models

The key adult emergent themes were “Design,” “Temporal Representation of PA,” “Engagement,” and “Motivation,” with a few distinct subthemes (Figure 3). The higher order theme *Engagement* was linked to additional subthemes *Sex differences*, *Teacher involvement*, and *Intervention*. Adults described characteristics similar to those used to construct both abstract (7/15, 47%) and graphical (8/15, 53%) model representations. Adults placed emphasis on making the 3D models attractive and recognizable but also something that challenges children’s and adolescents’ numeracy skills to work within the school curriculum:

Bar charts and graphs, that’s a big part of numeracy, so if you could maybe like make a physical graph...and it would go up in bars every day

TF8

Moreover, 1 parent added that a link between the 3D model and a recommended goal for physical activity could help encourage youths to achieve greater PAL:

...you know...maybe there’s strips [on the model] and each one, I don’t know if you reach the sort of recommended goal you get like another strip or something [on the model].

PF5

Similar to youths, adults preferred a week (12/15, 80%) of physical activity data represented on the model, as this was thought to have greater potential to visually guide youths, creating more awareness of their physical activity behaviors than a day (3/15, 20%) or month (1/15, 7%). Furthermore, some adults emphasized that changing the color (3/15, 20%) of lines on the models could visually aid participants in distinguishing the different days. The majority (13/15, 87%) of adults believed that if youths received and compared new models over time, this would act as a strong motivation for increased engagement in physical activity:

They [youths] could see at the end of five or six weeks...they could place their models and compare them thenthen you’re definitely motivating them [youths].

TM4

Furthermore, some teachers (4/8, 50%) reported that receiving their own 3D models would act as an additional competition and potential motivation for the pupils. However, some adults (7/15, 47%) expressed that the 3D models may ostracize youths from others if they underachieved in physical activity.

they [the models] might ostracize them...you know where they might go...no that’s too painful because, they’re going to get a better [model].

PF5

Adults perceived both positives (14/15, 93%) and negatives (14/15, 93%) for participants’ engagement with the concept of 3D printing physical activity. Positives included that the use of new technology (ie, 3D printers) would create awareness of current technological advances, with negative responses highlighting concerns about potential disruptions to teaching during lesson time. Moreover, some adults (8/15, 53%) believed that there would be no differences in how boys and girls engaged with the models, although 5 adults (33%, 5/15) highlighted that the girls may be more reflective and the boys more competitive:

I think the boys are more upfront and “what did you get and what did you get, let me see yours [the model]”, whereas I think a few of the girls would do that but the rest I think would do it more reflective when they’re on their own.

TF5

## Discussion

### Principal Findings

The aims of the research were first, to formatively elicit children’s, adolescents’, teachers’, and parents’ perceptions of physical activity data when represented as 3D-printed objects and their personal designs and to examine the perceived benefits and barriers to intervention participation. This research extends from that of previous studies that have implemented formative research techniques to inform the development of school-based interventions [47,59]. The second aim of the study was to use the formative data to inform the design of 3D models of physical activity to enhance youths’ understanding, awareness, and PAL.

The data indicated that youths can conceptualize physical activity data represented as a 3D object. This ability to detect and mentally represent a relationship between a symbol (ie, 3D object) and its referent (ie, physical activity) is known as representational insight [60]. However, the visual nature of the models does not always guarantee representational insight and its relation to the intended use [60]. For example, adolescents in this study showed greater ability to analyze and critique the physical activity behaviors represented on the prototype models. Adolescents could highlight, in some detail, differences in low and high PAL and how this related to their own and others’ personal habits. These differences between adolescents and children could be explained by a greater age-related cognitive ability in adolescents [61]. However, differences in cognitive ability may be less influential, as evidence suggests that visualizations help make complex information more accessible and cognitively tractable [60]. More specifically, previous research supports the use of tangible objects to stimulate youths’ intellectual development as they support a more natural way of learning [32,39,40,62,63], aligning with youths being regarded as *visual and tactile* learners [24]. For example, Gillet et al [32] investigated the use of 3D-printed enzyme molecules for teaching biology in youth, reporting that the tangible models provided a natural and intuitive mechanism for manipulation, exploration, and a proprioceptive pathway for learning. Although these findings hold promise, given that youths recognize the relationship between the tangible visualization and its intended referent, which is a necessary condition for developing a visual learning tool, others argue that an isolated approach is not sufficient [60,64-66]; it is important that youths understand the meaning and importance of the concepts represented on the visualizations to enable increased awareness of their personal physical activity behaviors [60]. In this light, future research should consider investigating 3D-printed physical activity feedback conditions to include and exclude an additional classroom educational component on PAL to fully understand the benefits of the 3D model alone.

This study revealed that youths believed the 3D models would act as a motivational tool to enhance their own PAL and that of their peers. Indeed, previous research suggests that school-based interventions that promote youths’ physical activity with the presence of peers significantly increase their motivation for physical activity [67] as well as their enjoyment [68,69], intensity [70], and engagement in out-of-school physical activity [71]. Furthermore, the majority of primary school children expressed that the 3D models would introduce competition between classmates, motivating them to engage in more physical activity. It has been argued that competition between children can be healthy if it provides feedback about performance and improvements, where children can learn about themselves, and the sole or primary objective is not about winning [72]. Conversely, adolescents placed more emphasis on how they would be motivated by beating their own personal model from the week before rather than comparing with others. These differences between youths could be, in part, explained by the adolescents’ greater understanding of the concept of effort in the physical domain [73] and applied ability to think independently, fostering enhanced self-evaluation skills that are important for preparation into adulthood [74]. Parents and teachers also agreed that the models would help motivate children and adolescents, allowing them to compare the models over time. Adults highlighted that boys may take a more competitive approach than girls who may engage in more reflective thoughts about the 3D models. Indeed, evidence suggests that young males engage in more individualistic competition than female counterparts [75]. Contrary to this, Bjorkqvist [76] found that girls use subtler, more indirect strategies for competition than boys from childhood to adulthood. Adolescents also displayed concerns that they might be perceived as inactive by their significant others, a consensus that was supported by the adults. Similar concerns have been raised when using digital fish avatars, the growth and emotional state of which is dependent on the participant’s PAL, with participants reporting being discouraged from using the app if they saw that the fish avatar was unhappy [77]. Therefore, monitoring how youths personally evaluate models displaying low PAL, and their support and interactions with significant others should be considered further. Beyond the scope of this study, it is pertinent to note that further research is also required to adapt these models to other populations and cultures, with the current results suggesting that children with special educational needs may misinterpret the models with negative health consequences for PAL, such as increased engagement with computer-based behaviors.

For the adults, the tangibility of being able to hold something that participants have created was perceived as original and personalized. Adults expressed that the tactile forms of information would interest youths and encourage them to purposely think about the importance of physical activity, as previously identified by Mackintosh et al [18]. Furthermore, they also believed that the 3D models could act as a material reward or medal representing achieved physical activity:

[something children and adolescents] could put [the models] up on their wall when they get them.

PM7

Indeed, much research suggests that material rewards are cherished more than virtual rewards [28], as a result of their higher visibility and low replication possibility [29,30]. Incentive-based interventions to encourage youth to take part in more physical activities have been shown to have promising effects [43,78], although findings have been mixed regarding sustained behavior change following removal of incentives [79]. Sport capitalizes on this incentive form of reward system with physical medals and trophies being presented to individuals. However, although these rewards focus on the completion of certain fitness or sports goals, they do not embody any personal data or represent the active self [80]. Khot et al [80] do, however, note that there is a learning value to be gained from *blending* rewards and representations to create more personalized and meaningful data. This concept is supported by findings from *Pokémon GO*, where children and adolescents can create and identify themselves with a visual avatar surrounded by recognizable characters (eg, Pikachu) in a socially networked system, which was associated with significant increases in physical activity in both age groups [81].

The current utilization of Play-Doh enabled youths to creatively explore, adapt, and develop their personal 3D model creations. This relatively inexpensive form of design prototyping has been used previously with malleable materials and is effective for brainstorming new ideas and designs from which high-tech prototypes emerge [20]. Our study’s findings revealed that children and adolescents preferred different types of 3D model design, leading to the development of 2 age-specific 3D models of physical activity. For children, a preference for a combination of both abstract (43%, 12/27) and graphical (54%, 15/27) models was demonstrated, most commonly expressed as Play-Doh models of flower- or sun-like shapes. However, to avoid any potential sex bias resulting in boys dissociating with a flower-shaped 3D model, the more neutral sun-shaped 3D model design was chosen for further development. Interestingly, a majority of adolescents (67%, 28/42) showed a preference through Play-Doh models for a simple bar chart design. However, with regard to the 2 different age-specific 3D models identified, there is limited literature as to whether the mapping of data should be abstract or graphical. Abstract data allow users to be more curious and speculative, whereas graphical representations provide more direct and comprehensive representations of data. Davis et al [82] suggest that more informative feedback provides greater opportunities to learn and improve performance. Indeed, it has been shown that tangible bar charts have benefits for information recall when compared with digital visualizations [83]. Contrary to this, more abstract methods of feedback may provide more positive engagement and support [84]. Anderson et al [85] also suggest that abstract visualizations increased motivation to achieve higher PAL in adults. Subsequently, adults believed that both methods of mapping a week of physical activity data were equally important, adding that presenting daily physical activity could potentially *overwhelm* the children and adolescents with data. As Khot et al [80] pointed out, embedding too much data can make the material model less readable, but on the other hand, with too little data, the model loses its intended purpose.

Although physical activity recommendations for youths are set to advise them on how to achieve an active lifestyle and create awareness of the important health benefits, few children were able to identify the UK-recommended amount of physical activity. Children’s interpretations of how much physical activity they should achieve were largely based on *how much sport* or *how many different sports* they could complete per day (eg, football, rugby, netball, and running), aligning with previous research findings [86]. In comparison, the adolescent group showed greater knowledge of the government guidelines, but this may have been influenced by the ongoing Sport Wales initiative 5x60 [58] implemented at the time of the study and aimed at encouraging youths to engage in 60 min of MVPA every day within school. However, it was evident that neither children nor adolescents were able to associate their understanding of the UK government recommendations with the intensity levels of MVPA, which highlights the need to promote youths’ knowledge of government recommendations, as reported by Mackintosh et al [18]. As aforementioned, tangible interfaces may offer a more playful learning experience [39] and natural interaction than other learning interfaces [87-89], suggesting that the tangibility of data may benefit children’s and adolescents’ learning [62]. As 1 parent expressed, creating a recommended goal for the youths on the model could be beneficial. Therefore, using a goal-setting strategy [41] and structurally developing the government recommendation into a tangible goal on the model may not only help in developing children’s and adolescents’ understanding of the government recommendations of 60 min of MVPA but also motivate youths to increase their PAL.

### Limitations

One of the major strengths of this study is its originality; however, this also highlights the paucity of other supporting research for this age group and that further investigation is warranted on this tangible form of data representation. Research should focus on the relative effectiveness of different types of 3D-printed visualizations of physical activity for the promotion of active learning in youths and as a means of strengthening the articulation of such initiatives with public health guidelines (ie, 60 min of MVPA) to enhance understanding and increase the motivation and engagement of youths in sustained physical activity.

### Conclusions

This formative study provides insight into the utilization of tangible 3D-printed objects displaying physical activity as a tool to benefit children and adolescents. The findings demonstrate how youths actively and enthusiastically engaged with the concept of 3D objects of physical activity and felt it could not only enhance their understanding of, but motivate them to increase, their PAL. From pupils’ Play-Doh model outputs, 2 age-specific 3D models representing weekly physical activity data were developed. The results of the formative research support the design of school-based physical activity interventions that utilize 3D printing of youths’ personal data as a unique strategy to promote their engagement in physical activity.

## Abbreviations

LED: light-emitting diode
PAL: physical activity levels
MVPA: moderate-to-vigorous physical activity
SES: socioeconomic status
YPAPM: Youth Physical Activity Promotion Model
